# Quantitative imaging for ^177^Lu-PSMA treatment response monitoring and dosimetry

**DOI:** 10.3389/fnume.2023.1291253

**Published:** 2023-12-14

**Authors:** Catherine Meyer, Laszlo Szidonya, Celeste Winters, Anna Mench, Nadine Mallak, Erik Mittra

**Affiliations:** Department of Diagnostic Radiology, Oregon Health & Science University, Portland, OR, United States

**Keywords:** SPECT/CT, lutetium-177, PSMA, prostate cancer, dosimetry

## Abstract

PSMA-targeted radiopharmaceutical therapy is an established treatment option for metastatic castration-resistant prostate cancer (mCRPC). However, response rates and duration using ^177^Lu-PSMA-617 vary considerably between patients. Quantitative ^177^Lu SPECT imaging is one approach that may be leveraged to more closely monitor inter-cycle response, as well as patient-specific absorbed doses. In this work, we describe our experience implementing quantitative imaging throughout the course of ^177^Lu-PSMA treatment, including serial SPECT imaging to monitor response and for individualized dosimetry. We also describe our imaging protocols and dose calculation workflows for 3D voxelized patient-specific organ and tumor dosimetry, including a review of the current landscape and efforts towards harmonized dosimetry.

## 1. Introduction

Radiopharmaceutical therapy (RPT) targeting the prostate specific membrane antigen (PSMA) has emerged as a safe and effective treatment for prostate cancer. ^177^Lu-PSMA-617 (^177^Lu-vipivotide tetraxetan, Pluvicto®; Novartis) received FDA approval in 2022 after showing significantly improved overall survival and progression-free survival compared to standard of care in the VISION trial (1, 2). The eligibility criteria for the VISION trial are typically followed for patient selection in clinical practice, defined by the presence of PSMA uptake higher than background liver in measurable lesions (1, 3). As currently practiced, RPT is a major step towards precision medicine through the use of imaging agents that allow the mapping of the molecular target expression in the tumor. However, it has been shown that not all eligible patients respond equally to treatment. The most established predictive factor so far is the degree of PSMA expression in the tumor, with higher tumor uptake (quantified by whole body tumor SUV_mean_) associated with better response to treatment, suggesting the potential for further optimization of patient selection (4–6).

In addition to the β-particle emission which causes DNA damage resulting in the primary therapeutic effect, ^177^Lu γ-emissions allow post-therapy imaging and SPECT-based dosimetry. Post-^177^Lu-PSMA SPECT/CT offers the unique opportunity of assessment of response to treatment while the therapy is ongoing, without the need to administer a separate diagnostic radiopharmaceutical. The standard protocol of ^177^Lu-PSMA consists of up to 6 cycles of 200 mCi every 6 weeks. However, emerging data suggest that early progression detected on post-therapy imaging (at 12 weeks, or post-cycle 3) is predictive of poor response, and may therefore be used as a biomarker that can help avoid unnecessary cycles, mitigate potential side effects, and allow other therapies to be started sooner (7).

Personalized dosimetry is gaining increased attention with the promise of tailoring RPTs to individual patients. Emerging evidence establishes a positive correlation between elevated uptake on pre-therapy PET and post-therapy dosimetry calculations of absorbed dose, as well as a positive correlation between the tumor absorbed dose and treatment response (8, 9). In addition to optimizing the therapeutic effect, dosimetry may help mitigate potential radiation-related toxicities.

## 2. Quantitative SPECT imaging

The goal of quantitative imaging is to accurately represent the activity distribution of the radionuclide, which impacts both radiation dose and SUV measurements. Unlike PET images, which are routinely displayed in units of SUV, SPECT images are typically displayed with voxel units of counts and must be calibrated to units of absolute activity concentration for quantitative applications and SUV calculation. Additionally, SPECT-based activity measurements at multiple time points are used to calculate absorbed dose, which strongly depends on the accuracy of the quantitative SPECT images.

Quantitative ^177^Lu SPECT protocols are not currently harmonized across institutions, but there is an increasing number of publications addressing quantitative protocol design (10–12). To convert from voxel counts to activity concentration, a scanner-specific sensitivity factor, which can be derived from phantom studies, must be applied. For the ^177^Lu SPECT images included in this report, calibration factors for two Siemens Intevo Bold SPECT/CT scanners were determined using a phantom filled with a known activity of ^177^Lu and scanned using the clinical ^177^Lu protocol. Clinically, patients are scanned from vertex to mid-thighs (3 bed positions) utilizing a 20% photopeak window centered on 208 keV, a 10% lower scatter window, and 128 projections (see Supplementary Table S1 for complete details). A vendor-neutral iterative reconstruction (SPECTRA Quant, MIM Software, Cleveland, Ohio) was used to incorporate attenuation correction, scatter correction, resolution recovery, and the phantom-derived scanner-specific calibration factors.

Ideally, best practices for image quantification will also account for count loss due to partial volume effects. A common correction method is to apply recovery coefficients (RC), which are size-dependent count loss correction factors that can be estimated using phantoms with spherical inserts of varying size. However, RCs do not only depend on object size, so are ideally calculated using more faithful anatomical representations (13, 14).

## 3. SPECT imaging for treatment monitoring

All patients' treatment eligibility is first determined by a PSMA PET scan to confirm target expression. Additionally, our institution performs quantitative SPECT/CT imaging after the first and third treatment cycles to monitor response while treatment is ongoing. Patients referred for treatment with ^177^Lu-PSMA undergo a quantitative SPECT/CT scan from vertex to mid-thighs 24 h following radiopharmaceutical administration for cycle 1. This scan serves as a new baseline for treatment as there may have been changes in the disease burden (typically progression) between the initial PET and onset of treatment. A second SPECT/CT scan is acquired 24 h after cycle 3 to monitor interim treatment response halfway through the standard dosing regimen. Based on the clinical need, patients may undergo additional SPECT/CTs after subsequent treatment cycles. These post-therapy scans provide both qualitative and, with appropriate scanner calibrations as detailed above, quantitative information to aid in disease management, along with clinical measures such as serum prostate-specific antigen (PSA) values, and patient symptoms.

The best way to quantify the total tumor volume (TTV) using the initial PET and post-therapy SPECT scans is not yet established. As such, we investigated 3 different methods: a global SUV threshold of 3, a PERCIST-based liver-derived threshold, and a deep learning assisted whole body segmentation solution from MIM Software (Cleveland, Ohio). A user-defined global SUV threshold of 3 was selected based on previously published studies of tumor burden quantification (7, 8, 15). The PERCIST liver-derived SUV threshold was determined by placing a representative 3 cm spherical ROI in the right lobe of normal liver and the threshold was calculated as: 1.5*liver_mean + 2*liver_SD (16). For both segmentation approaches, physiologic PSMA uptake is removed manually by a nuclear medicine physician. The deep learning assisted approach utilizes a global SUV threshold of 3 (with the exception of liver lesions) to provide an initial segmentation. Subsequently, this method utilizes deep learning to generate normal structures on CT to categorize physiological uptake for removal (17, 18). Liver lesion segmentation is performed similar to the PERCIST approach based on an automatically placed ROI for normal liver tissue. TTV was interpreted alongside patient PSA kinetics.

**Figure 1:**
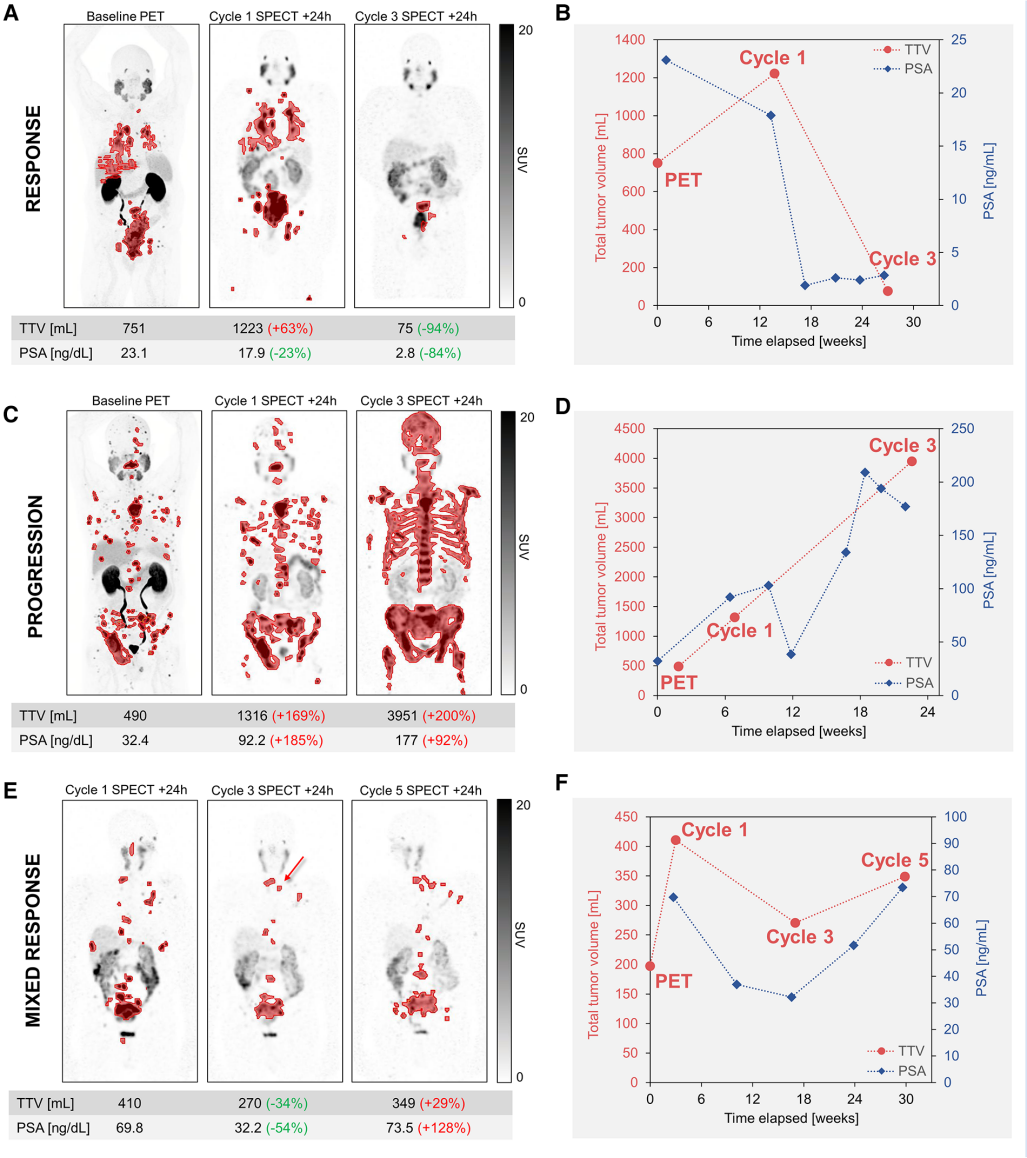
Serial quantitative imaging showing baseline PET (^18^F-DCFPyL) and post-therapy SPECT images acquired 24 h after cycles 1 and 3 of ^177^Lu-PSMA. Segmented lesions are shown in red, and the relative changes in TTV and PSA for each time point are calculated relative to the prior imaging time point. TTV and additional serum PSA values are plotted to visualize tumor burden and PSA kinetics. Maximum-intensity projections are shown for representative patients exhibiting response (**A,B**) or progression (**C,D**). For a patient exhibiting a mixed response (**E,F**), post-therapy SPECT images are shown for cycles 1, 3, and 5. Despite the drop in PSA and the resolution or improvement of the majority of metastatic lesions at cycle 3, post-therapy imaging allowed the early detection of a few new lesions (red arrow), consistent with mixed response, followed by further progression. Additional new lesions were more evident on the SPECT/CT than the SPECT MIP and are not shown. All SPECT images are equally scaled. TTV, total tumor volume; PSA, prostate-specific antigen.

In our experience, TTV based on a global SUV threshold of 3 with manual removal of physiological PSMA uptake was found to be the most satisfactory approach based on visual analysis of lesion coverage and exclusion of physiological uptake. This approach is shown for representative patients exhibiting response, progression, and a mixed response (Figure 1). Comparisons of TTV segmentation methods are included in Supplementary Figures S1–S3, but a systematic evaluation of the validity of segmentation methods is beyond the scope of this report.

As seen in Figure 1, the TTV measured by post-therapy SPECT imaging is well-correlated with the biochemical PSA response. In a representative patient who responded well to ^177^Lu-PSMA, as reflected by >50% decline in PSA, there was a dramatic 94% decrease in TTV and 84% decrease in PSA at cycle 3 relative to cycle 1 (Figure 1A). SPECT/CT at cycle 1 serves as a baseline scan for RPT, as it is not uncommon to see disease progression between the baseline PSMA PET and the start of the therapy, as is the case shown in Figure 1C. In this case, biochemical progression and increased TTV were both observed at cycle 3 relative to the baseline scan at cycle 1 (Figure 1C). For a patient regarded as a mixed responder, while there was a 34% decrease in TTV at cycle 3, SPECT imaging allowed detection of a few new lesions relative to cycle 1, and later imaging after cycle 5 revealed progression by all included metrics (Figure 1E). In addition to the SPECT findings, the CT component of the SPECT/CT is also used to identify developing or worsening PSMA negative disease, which remains untreated by ^177^Lu-PSMA. Since flare phenomenon after the first cycle of treatment is possible, though thought to be uncommon (19), we believe that response assessment at cycle 2 may be too early. It is prudent to wait for confirmation of tumor progression at cycle 3 before a change in management; this approach however needs confirmation in future prospective studies. These are only representative cases and larger cohorts are needed to establish the appropriateness of different TTV segmentation approaches and the added prognostic value of an image-based TTV metric.

## 4. Dosimetry

Despite the potential benefit of personalized therapy, RPTs are currently administered at a fixed injected activity. While this approach led to the FDA approval of ^177^Lu-PSMA-617, more attention is shifting to individualized treatment planning that reflects the patient-specific disease burden and clearance kinetics of the RPT agent.

### 4.1. Clinical implementation

While performing dosimetry calculations for every RPT patient at every cycle would provide the most comprehensive patient dose estimation over the course of treatment, this is not practical with current methods. Rather, dosimetry is often performed for the first cycle only, the results of which are extrapolated to subsequent cycles. Another important clinical question is which patients would benefit most from dosimetry. Currently, our institution performs dosimetry in select patients (five, to date) who present with a disease pattern or clinical factors for which a potential concern might exist for treatment with the standard activity dosing regimen. Examples include patients with significant PSMA uptake in normal organs and patients with impaired kidney function or a single kidney. In addition, patients with heterogeneous uptake in lesions may also benefit for the purpose of predicting treatment efficacy. Together with the therapy, the dosimetry work is billed to the patient's insurance using several CPT codes for both imaging and the associated calculations (20).

Using the standard injected activity, individualized dosimetry calculations are carried out for the first cycle of treatment and extrapolated to future cycles. Patients undergo serial quantitative SPECT/CT imaging from the vertex to the mid-thighs at 4 time points (4, 24, 48, and 72 h) following ^177^Lu-PSMA administration. It is common to acquire a minimum of 3 time points for sampling the biodistribution, with time points selected based on effective clearance times for Lu-PSMA and practical considerations of the clinic schedule (10, 21). Image acquisition details and quantification protocols are carried out as described above (Section 2).

Image and dosimetry analysis are performed using commercial software (MIM Software, Cleveland, Ohio). First, the following organs are contoured on CT images using a deep learning segmentation algorithm: kidneys, parotid glands, liver, and lungs. Organ contours are adjusted manually and lumbar vertebrae (without metastasis) are segmented for bone marrow dose estimation. Tumor lesions are segmented on the 24 h SPECT images using a patient-specific SUV threshold and propagated to the other SPECT time points based on automated ROI-specific rigid registrations. Unlike the standardized SUV threshold of 3 utilized for the post-therapy SPECT/CTs, the SUV threshold for tumor dosimetry is selected on an individual patient basis by an experienced nuclear medicine physician. Once the desired tumor targets and organs are contoured, the activity concentrations in each region over time are used to create voxel-level time-activity curves, generating dose-volume histograms for clinical interpretation.

### 4.2. ^177^Lu-PSMA dosimetry example

**Figure 2:**
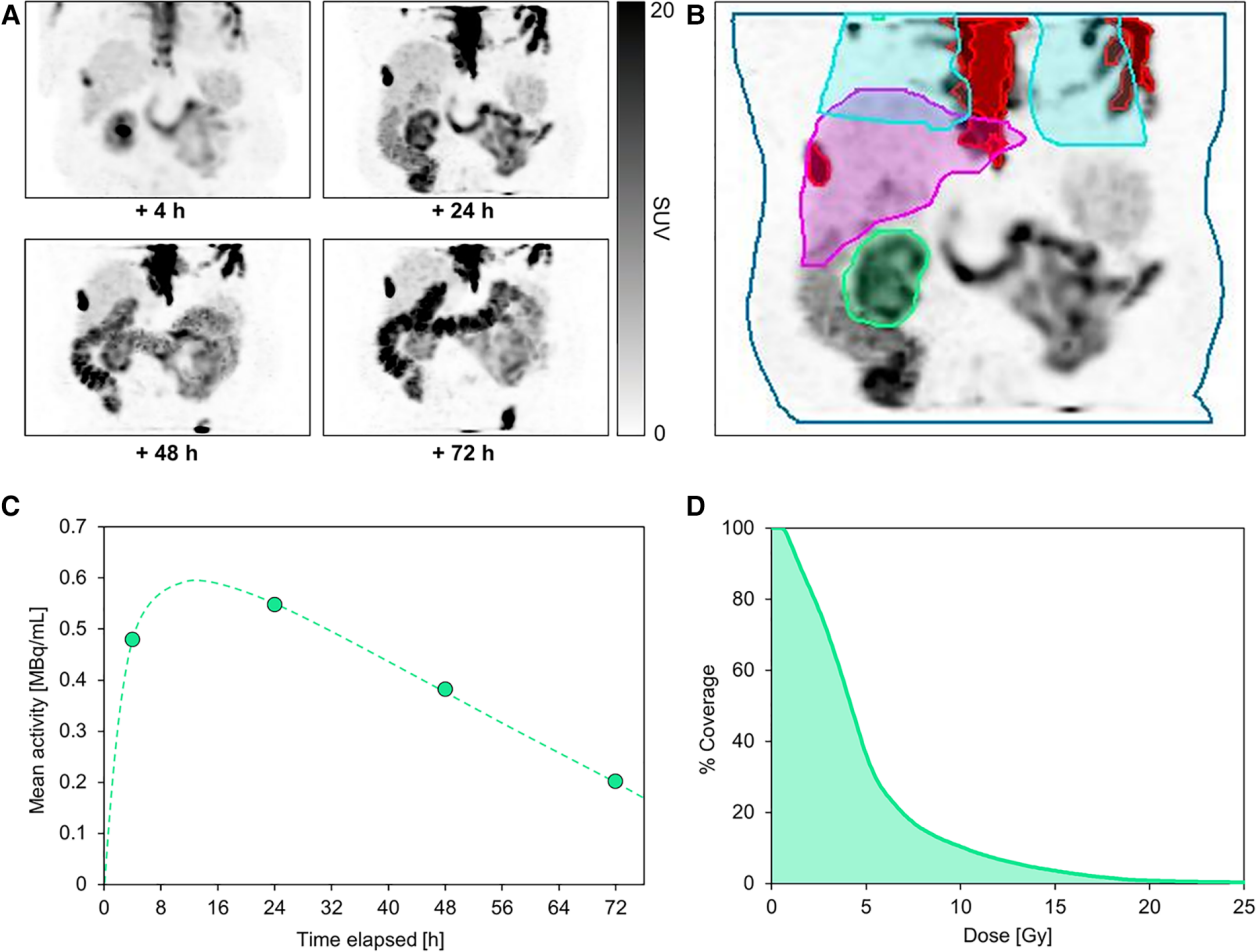
Serial post-therapy SPECT maximum-intensity projections acquired at 4, 24, 48, and 72 h following administration of the first cycle of ^177^Lu-PSMA (**A**). This patient underwent multi-bed SPECT/CT imaging from vertex to mid-thighs 4 h after treatment, but on subsequent imaging days the field of view was limited to a single bed centered over the kidney to reduce scan time for patient comfort. All SPECT images are equally scaled. Normal organ and segmented lesion contours are shown on the +24 h SPECT image (**B**), including kidney (green), liver (magenta), partial lungs (blue), and lesions in the field of view (red). Absorbed dose to parotid glands was not computed due to the limited field of view at later imaging time points. A representative kidney time-activity curve based on quantitative SPECT-derived mean activity measurements is shown for the kidney over the course of the treatment (**C**). The dose-volume histogram for the kidney (**D**) illustrates the estimated dose coverage within the kidney volume.

The following case is of a 76-year-old patient who was selected for dosimetry due to having a single kidney with borderline baseline function (creatinine 1.6 mg/dl, GFR 44 ml/min). He underwent a series of 4 post-therapy imaging scans up to 72 h after the first treatment. Figure 2 shows the patient images, as well as the organ and lesion contours used for dose estimation.

The dosimetry analysis revealed a mean absorbed dose to the kidney of 5.15 ± 4.07 Gy. The absorbed doses to liver, bone marrow, and lungs were 1.68 ± 1.10 Gy, 0.27 ± 0.07 Gy, and 1.45 ± 1.46 Gy, respectively. In total, 10 lesions were contoured with an overall mean tumor dose of 6.9 ± 7.1 Gy (ranging from 1.1 to 9.7 Gy among individual lesions). Assuming that the kidney dose remains constant over each of the 6 cycles, the total cumulative kidney dose would exceed 30 Gy; whereas a commonly-cited historical tolerance dose limit is 23 Gy (22). Despite growing evidence that doses over 23 Gy may be tolerable, the potential dose limitation to the kidney in this case must be comprehensively considered alongside risk factors and prognosis, including a potential activity de-escalation in future cycles or a reduced number of cycles (23–25). To date in our practice, dosimetry results are used as one quantitative metric among many clinical measures (such as other treatment options, age, comorbidities, life expectancy, etc.) to evaluate risk to patients. Treatment decisions are made collectively between nuclear medicine physicians and referring providers, often in a multidisciplinary setting. In the future, ^177^Lu-PSMA may be introduced earlier in the treatment paradigm for patients with longer life expectancy and other treatment options, further increasing the impact of performing dosimetry.

## 5. Discussion

While the current clinical practice of ^177^Lu-PSMA therapy represents an important milestone for patients with mCRPC, the safety profile and treatment efficacy may be improved through the use of quantitative SPECT imaging for response assessment and personalized dosimetry.

### 5.1. SPECT quantification subtleties

Although absorbed dose and SUV estimation are possible through quantitative SPECT calibrations, there are many factors, both controllable and uncontrollable, that have downstream effects on quantification of counts, including scanner model, reconstruction parameters, protocol settings, and patient-dependent factors. For example, orbit radius, collimator, matrix size, and lesion/organ location may all affect resolution, and lower resolution introduces a negative bias on measured activity (12). It should also be considered that the SPECT resolution exceeds the ^177^Lu β-particle pathlength, which may be important when evaluating spatial dose heterogeneity (26).

### 5.2. Organ and lesion segmentation

While normal organ segmentation based on CT images is relatively straightforward, segmentation of smaller organs, such as the lacrimal glands, can be challenging. Segmentation of the PSMA-avid disease is an active area of research as interest in identifying imaging biomarkers and quantitative interim imaging is growing. While several publications have utilized an SUV threshold of 3 for both PSMA PET and SPECT images, as was exemplified in this report, general agreement in the field remains to be reached and larger validation studies are needed. While using a patient-specific SUV threshold (as was done for the example dosimetry case described above) represents a more personalized approach, it is also more subjective and less reproducible among users and institutions. Moreover, global thresholding approaches may be inadequate for segmentation of lesions with low level uptake, including lung, liver, and lymph node metastases, which may benefit from manual or automated lesion-specific contouring. To that end, segmentation presents an opportunity for artificial intelligence applications in identifying lesions, reducing segmentation time burden, and recognizing physiological uptake.

### 5.3. Dosimetry standardization

Currently, there is a lack of standardization with regards to the methodology and implementation of dosimetry for ^177^Lu-PSMA and RPT overall. One common approach is to conduct multiple time-point dosimetry for the first cycle of treatment followed by extrapolation of that dose through subsequent cycles. While simple extrapolation of the first cycle dose is straightforward, changes in tumor burden and clearance kinetics over the course of treatment may introduce inaccuracies in the extrapolation assumptions. Another approach for simplified dosimetry is to scale a patient-specific or population-based kinetics curve using activity measured from a single time point of imaging (27–30). Further, next-generation SPECT scanners, such as those incorporating cadmium zinc telluride detectors, are expected to increase feasibility of scanning patients for dosimetry, thanks to improvements in sensitivity, resolution, and scanning speed (31). As the body of literature for PSMA dosimetry grows, and more software tools and hardware advancements become available, attention should be directed towards dosimetry harmonization to ease clinical utilization.

### 5.4. Radiobiological considerations

Currently utilized organ tolerance dose limits for RPT are based on decades of clinical experience and published data specific to external beam radiation therapy dose-effect relationships (22, 25). While applying these limits for RPT is considered a conservative approach, it is known that the underlying radiobiological effects for RPT are different due to the radiation type, systemic heterogeneous dose delivery, and a decreasing time-dependent dose rate (32, 33). Therefore, the use of these tolerance dose limits must be coupled with consideration of risk factors and prognosis, including disease stage, previous treatments, life expectancy, and existing comorbidities. Ongoing research, including evaluations of long-term toxicities, is needed to establish appropriate dose limits for RPT.

Of note, most current dosimetry practices are operating on the basis of cautiously de-escalating treatment activity in the case of exceeding organ tolerance dose limits, not on the opportunity to increase the injected activity to optimize the anti-tumoral therapeutic response. While the latter is currently limited by the FDA-label, manufacturing practices, and insurance reimbursement, this may change in the future as lesional dosimetry is a fast-growing area of research generating increasing evidence that absorbed doses are correlated to treatment response (8, 34, 35).

### 5.5. Clinical implementation

Beyond the technical factors described thus far, there are additional clinical challenges to adopting these quantitative strategies, including the increased burden of additional scans (both on patients and a busy clinic), the support and expertise of medical physicists, a lack of uniformity across institutions, and last but not least, billing/reimbursement (20). In our experience, logistics surrounding patient scheduling and the duration of the dosimetry SPECT scans are the leading challenges to clinical incorporation. However, quantitative imaging and dosimetry are rapidly evolving areas of our clinical practice and as the field moves in this direction, ease of clinical implementation and harmonization will become priorities. Furthermore, studies evaluating imaging metrics and tumor dose response relationships, as well as clinical trials including dosimetric analysis are critical. Such studies will help elucidate the role of these quantitative measures relative to patient response, thereby driving the field towards more personalized disease treatment and optimized patient outcomes.

## 6. Conclusion

In this work we report our experience to date incorporating quantitative imaging into our ^177^Lu-PSMA theranostics practice. Serial ^177^Lu SPECT/CT imaging throughout the course of treatment enables interim assessment of quantitative tumor burden imaging metrics, through which early detection of progression can be confirmed to aid in clinical decision-making. Further, serial quantitative imaging can be used for patient-specific estimates of absorbed doses to normal organs and target lesions as a means of evaluating potential side-effects and quantifying the therapeutic efficacy. Longitudinal studies with larger cohorts are needed to evaluate the potential predictive value of baseline and interim ^177^Lu-PSMA SPECT images, as well as absorbed doses, relative to treatment outcomes and progression-free survival.

## Data Availability

The raw data supporting the conclusions of this article will be made available by the authors, without undue reservation.
