# Supplementary material for: Quantitative imaging for 177Lu-PSMA treatment response monitoring and dosimetry
Source: Front Nucl Med. 2023 Dec 14;3:1291253. doi: 10.3389/fnume.2023.1291253 (PMC11440845; doi:10.3389/fnume.2023.1291253)
Supplement: Supplementary file 1 [file Datasheet1.docx]

Supplementary Material

**SUPPLEMENTARY TABLES**

**Supplementary Table 1.** ^177^Lu SPECT imaging protocol parameters

| Scanner | Siemens Intevo Bold |
| --- | --- |
| Collimators | Medium energy |
| Photopeak window | 208 keV +/- 10% (187 – 229 keV) |
| Scatter window | 10% lower scatter window (166 – 187 keV) |
| Projections | 128 (2 heads x 64 projections) |
| Frame time | 15 seconds dosimetry scans, 5 seconds post-therapy monitoring scans |
| Bed positions | 3, vertex to mid-thighs |
| Orbit | Auto-contouring |
| Reconstruction | OSEM (8 iterations, 16 subsets) |
| Corrections | Attenuation, scatter, resolution recovery |

**SUPPLEMENTARY FIGURES**

**
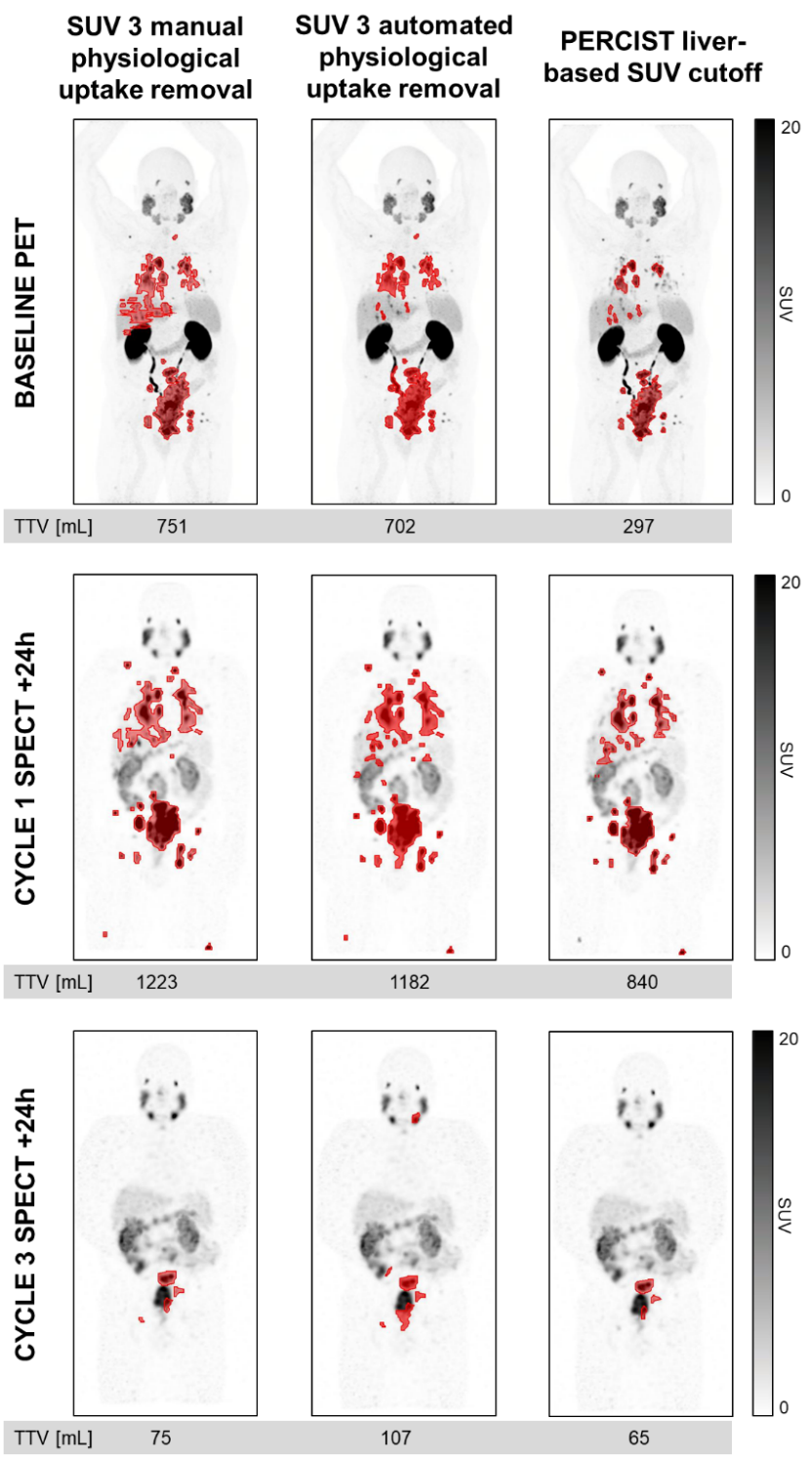
**

**Supplementary Figure 1.** Serial quantitative imaging showing baseline PET (^18^F-DCFPyL) and post-therapy SPECT images acquired 24h after cycles 1 and 3 of ^177^Lu-PSMA-617. Maximum-intensity projections are shown for a representative patient exhibiting response. Time delay between the initial PET and the 1^st^ cycle SPECT imaging was 13.7 weeks. The results of three different lesion segmentation methodologies are shown for comparison: SUV threshold of 3 with manual removal of physiological uptake, automated physiological uptake removal, and a liver-based cutoff. Segmented lesions are shown in red, and the relative changes in TTV are shown relative to the prior imaging time point. Initial PSA was 23.1 ng/dL, which decreased to 17.9 at cycle 1 and 2.8 by cycle 3. All images are equally scaled. TTV = total tumor volume.

*
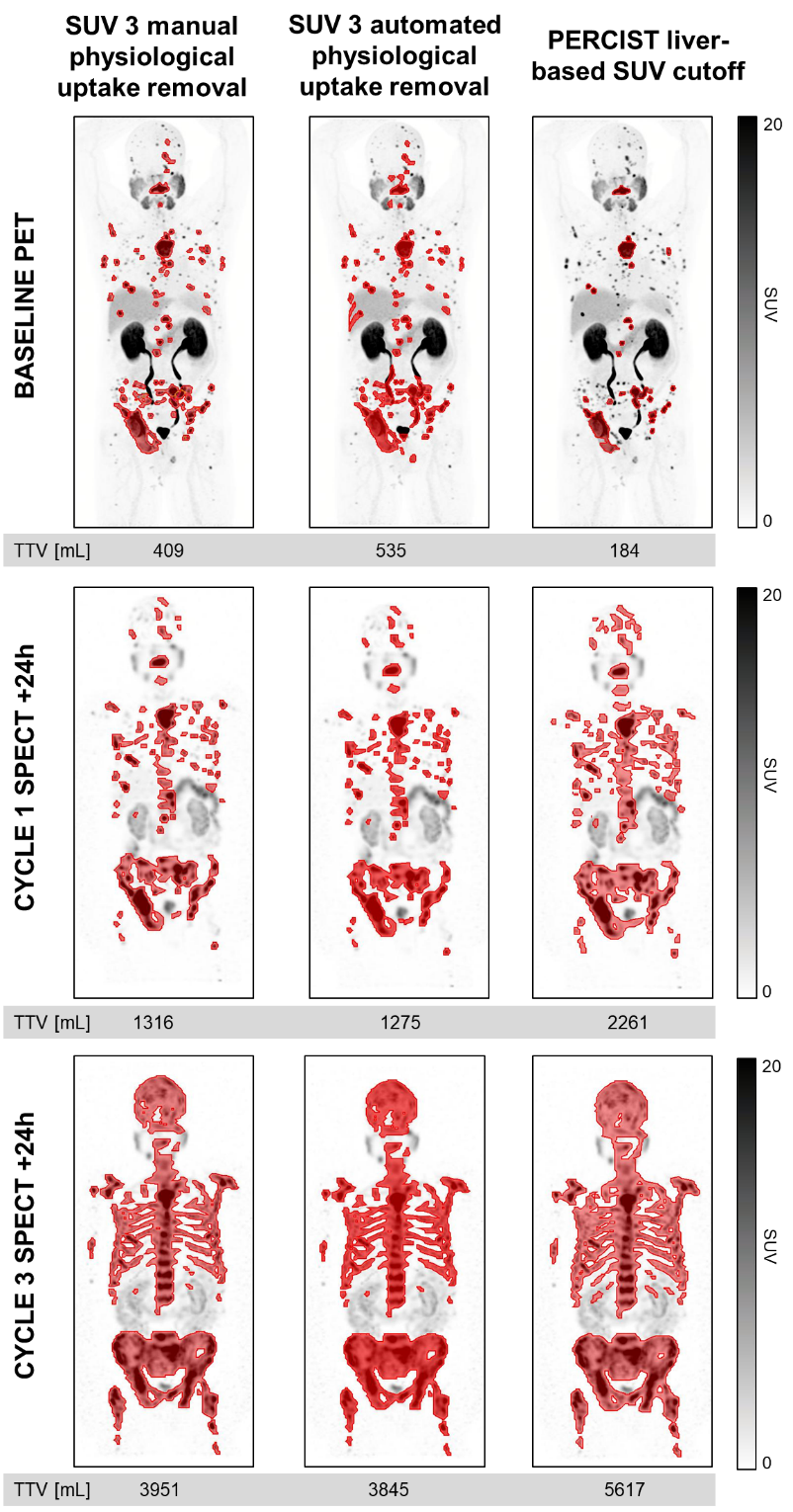
*

**Supplementary Figure 2.** Serial quantitative imaging showing baseline PET (^18^F-DCFPyL) and post-therapy SPECT images acquired 24h after cycles 1 and 3 of ^177^Lu-PSMA-617. Maximum-intensity projections are shown for a representative patient exhibiting progression. Time delay between the initial PET and the 1^st^ cycle SPECT imaging was 5 weeks. The results of three different lesion segmentation methodologies are shown for comparison: SUV threshold of 3 with manual removal of physiological uptake, automated physiological uptake removal, and a liver-based cutoff. Segmented lesions are shown in red, and the relative changes in TTV are shown relative to the prior imaging time point. Initial PSA was 32.4 ng/dL, which increased to 92.2 at cycle 1 and 177 by cycle 3. All images are equally scaled. TTV = total tumor volume.

**
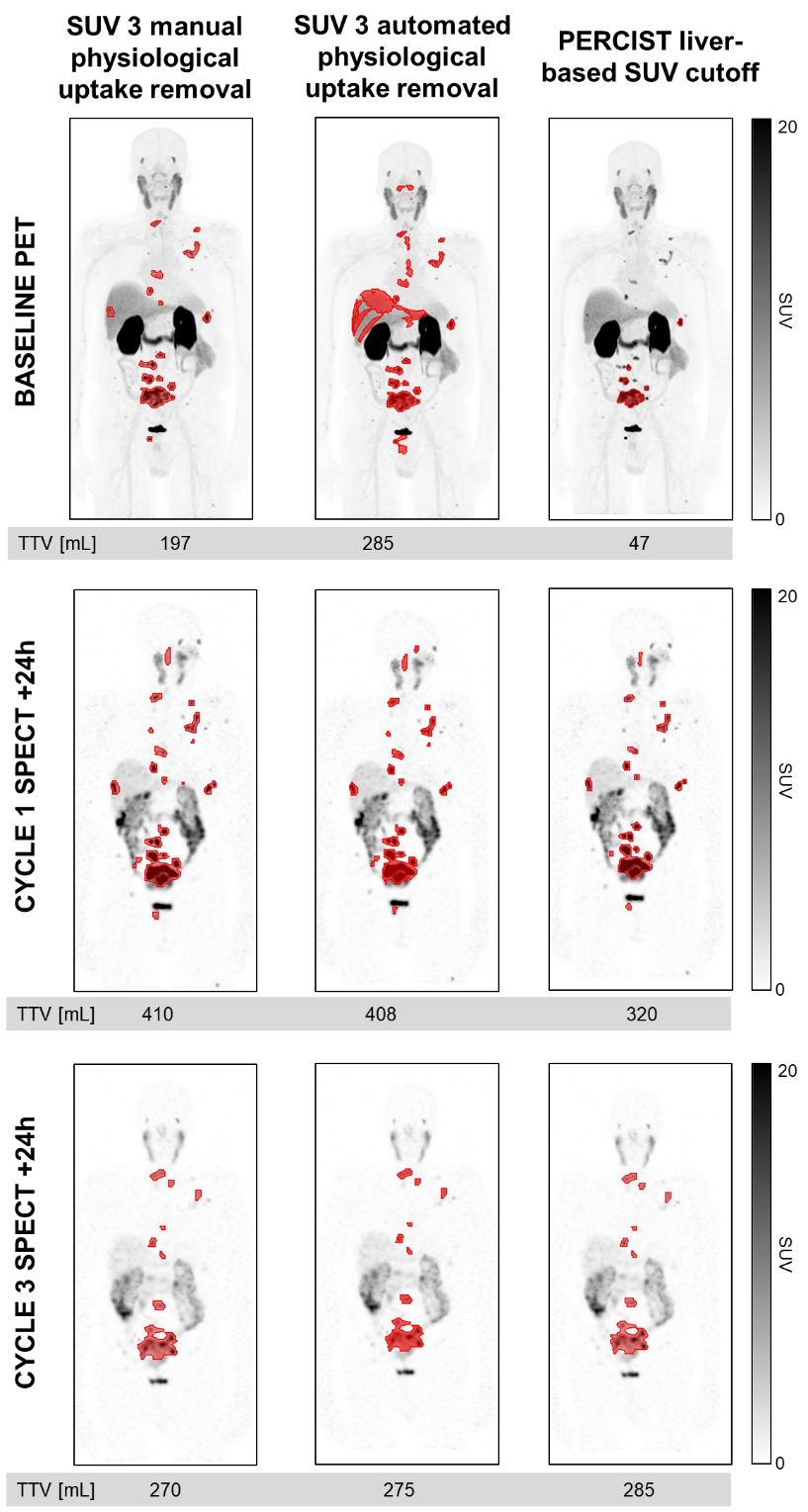
**

**Supplementary Figure 3.** Serial quantitative imaging showing baseline PET (^18^F-DCFPyL) and post-therapy SPECT images acquired 24h after cycles 1 and 3 of ^177^Lu-PSMA-617. Maximum-intensity projections are shown for a representative patient exhibiting mixed response. Time delay between the initial PET and the 1^st^ cycle SPECT imaging was 3 weeks. The results of three different lesion segmentation methodologies are shown for comparison: SUV threshold of 3 with manual removal of physiological uptake, automated physiological uptake removal, and a liver-based cutoff. Segmented lesions are shown in red, and the relative changes in TTV are shown relative to the prior imaging time point. Initial PSA was 69.8 ng/dL, which decreased to 32.2 at cycle 1 then rose to 73.5 by cycle 3. All images are equally scaled. TTV = total tumor volume.
